# Foot orthoses and physiotherapy in the treatment of patellofemoral pain syndrome: randomised clinical trial

**DOI:** 10.1136/bmj.a1735

**Published:** 2008-10-24

**Authors:** Natalie Collins, Kay Crossley, Elaine Beller, Ross Darnell, Thomas McPoil, Bill Vicenzino

**Affiliations:** 1 School of Health and Rehabilitation Sciences, University of Queensland, Brisbane, Australia 4072; 2 Melbourne School of Engineering, University of Melbourne, Australia; 3 Queensland Clinical Trials Centre, School of Population Health, University of Queensland; 4 Gait Research Laboratory, Department of Physical Therapies, Northern Arizona University, USA

## Abstract

**Objective** To compare the clinical efficacy of foot orthoses in the management of patellofemoral pain syndrome with flat inserts or physiotherapy, and to investigate the effectiveness of foot orthoses plus physiotherapy.

**Design** Prospective, single blind, randomised clinical trial.

**Setting** Single centre trial within a community setting in Brisbane, Australia.

**Participants** 179 participants (100 women) aged 18 to 40 years, with a clinical diagnosis of patellofemoral pain syndrome of greater than six weeks’ duration, who had no previous treatment with foot orthoses or physiotherapy in the preceding 12 months.

**Interventions** Six weeks of physiotherapist intervention with off the shelf foot orthoses, flat inserts, multimodal physiotherapy (patellofemoral joint mobilisation, patellar taping, quadriceps muscle retraining, and education), or foot orthoses plus physiotherapy.

**Main outcome measures** Global improvement, severity of usual and worst pain over the preceding week, anterior knee pain scale, and functional index questionnaire measured at 6, 12, and 52 weeks.

**Results** Foot orthoses produced improvement beyond that of flat inserts in the short term, notably at six weeks (relative risk reduction 0.66, 99% confidence interval 0.05 to 1.17; NNT 4 (99% confidence interval 2 to 51). No significant differences were found between foot orthoses and physiotherapy, or between physiotherapy and physiotherapy plus orthoses. All groups showed clinically meaningful improvements in primary outcomes over 52 weeks.

**Conclusion** While foot orthoses are superior to flat inserts according to participants’ overall perception, they are similar to physiotherapy and do not improve outcomes when added to physiotherapy in the short term management of patellofemoral pain. Given the long term improvement observed in all treatment groups, general practitioners may seek to hasten recovery by prescribing prefabricated orthoses.

**Trial registration** Australian Clinical Trials Registry ACTRN012605000463673 and ClinicalTrials.gov NCT00118521.

## Introduction

Patellofemoral pain syndrome, or idiopathic pain arising from the anterior knee,^1^ is one of the most common musculoskeletal presentations to general practice^2^ and sports medicine clinics.^3^ ^4^ ^5^ ^6^ ^7^ ^8^ In a retrospective survey of 2002 runners presenting to a sports medicine centre, patellofemoral pain syndrome accounted for 19% of running injuries,^9^ whereas a two year prospective cohort study reported onset of the syndrome in 9% of 282 students of physical education aged 17-21.^10^ The pain is characteristically provoked by activities such as squatting, stair walking, and running, and hence impacts on many aspects of daily life, including the ability to perform pain free exercise or work related activities. Patellofemoral pain syndrome can result in repeat visits to a doctor given its tendency towards chronicity, with 94% of patients continuing to experience pain up to four years after initial presentation and 25% reporting significant symptoms up to 20 years later.^11^

Despite the prevalence, chronicity, and impact of patellofemoral pain syndrome, several systematic reviews of interventions attest to a dearth of high quality research on management.^12^ ^13^ ^14^ ^15^ ^16^ ^17^ One study concluded that the available evidence at that time would lead the practitioner to implement a programme of education, stretching, and strengthening of the thigh muscles, and possibly foot orthoses.^15^ Subsequently a high quality randomised controlled trial found that a multimodal physiotherapy programme for six weeks^18^ was more effective than sham treatment: relative risk of noticeable improvement 3.39 (95% confidence interval 1.69 to 6.80).^19^ That study did not, however, compare physiotherapy with the control sham intervention beyond six weeks.

As an alternative or adjunct to physiotherapy, foot orthoses are commonly used to treat active people with patellofemoral pain syndrome. Recently, a systematic review of the clinical efficacy of foot orthoses identified two small clinical trials in people with patellofemoral pain syndrome.^20^ These studies suggest that foot orthoses may be of benefit.^21^ ^22^ No high quality randomised controlled trials have evaluated the use of foot orthoses for treating patellofemoral pain syndrome in the short or long term. Evidence to guide the use of foot orthoses for this common clinical condition is imperative considering the widespread use of foot orthoses and the lack of consensus and controversy surrounding their prescription.^23^ ^24^

We evaluated the short and long term clinical efficacy of prefabricated foot orthoses in the treatment of patellofemoral pain syndrome compared with flat inserts or physiotherapy alone, and evaluated whether orthoses improved the effects of physiotherapy. We hypothesised that foot orthoses would be superior to flat inserts and equivalent to physiotherapy and that the combination of foot orthoses and physiotherapy would be superior to physiotherapy alone.

## Methods

We carried out a pragmatic, single blind, randomised clinical trial in a community setting for 12 months. The methods have been described in detail previously.^25^

Volunteers from the greater Brisbane, Gold Coast, and Toowoomba regions of Queensland, Australia responded to advertisements in print media, radio and television media releases, noticeboards, and referrals from practitioners. Eligibility criteria were based on a previous clinical trial^19^: age 18-40 years; insidious onset of anterior knee or retropatellar pain of greater than six weeks’ duration and provoked by at least two of prolonged sitting or kneeling, squatting, running, hopping, or stair walking; tenderness on palpation of the patella, or pain with step down or double leg squat; and worst pain over the previous week of at least 30 mm on a 100 mm visual analogue scale. Exclusion criteria were concomitant injury or pain from the hip, lumbar spine, or other knee structures; previous knee surgery; patellofemoral instability; knee joint effusion; any foot condition that precluded use of foot orthoses; allergy to strapping tape; use of physiotherapy or foot orthoses within the previous year; or use of anti-inflammatory drugs.

### Protocol

To facilitate concealment of allocation, a blinded assessor not involved in the randomisation process determined eligibility. The randomisation sequence was drawn up and kept off site by an independent body, using a random number generator in blocks of eight with no stratification. Participants gave written informed consent and, after we had obtained baseline measures, were randomly assigned to receive one of four treatments: foot orthoses, flat inserts, physiotherapy, or foot orthoses plus physiotherapy. A research assistant communicated with the randomisation centre, participants, and project physiotherapists throughout the trial, thus ensuring that the assessor responsible for outcome measurement and data analysis remained blind to group allocation.

### Interventions

Interventions were administered by one of 17 registered physiotherapists who underwent training for each treatment protocol. Participants attended six appointments of 20-60 minutes’ duration over six weeks, after which they were encouraged to continue with a self management programme.

The intervention programmes have been detailed previously.^25^ In brief, participants assigned to foot orthoses received prefabricated, commercially available orthoses (Vasyli International), which were fitted to their shoes with comfort as a primary goal. These orthoses are customisable to some degree to optimise comfort through heat moulding and by adding wedge or heel raises. As a control for these orthoses we used flat inserts, manufactured from the same material (ethylenevinyl acetate) with identical covering fabric. These were of uniform thickness, with no inbuilt arch or wedging. Physiotherapy consisted of a combined therapy approach that has proved efficacious in patellofemoral pain syndrome^19^ and included patellar mobilisation, patellar taping, a progressive programme of vasti muscle retraining exercises with electromyographic biofeedback, hamstring and anterior hip stretches, hip external rotator retraining, and a home exercise programme. Participants assigned to orthoses plus physiotherapy received both interventions as described and had an extra appointment with the physiotherapist if more time was required for adequate delivery of all treatment components.

The participants were encouraged to continue exercise and activities that did not provoke their pain. The use of non-study interventions was discouraged throughout the trial, although over the counter drugs were permitted. Any cointerventions used for symptoms of patellofemoral pain syndrome, as well as any adverse effects arising from intervention, were recorded in diaries, reported to the research assistant, or detailed in an exit questionnaire.

### Outcomes

The blinded assessor carried out reliable and valid outcome measures^25^ ^26^ before randomisation (baseline) and at 6, 12, and 52 weeks after randomisation. The primary outcome measures were global improvement,^25^ severity of usual and worst pain over the preceding week, the anterior knee pain scale,^27^ and the functional index questionnaire.^28^ We measured global improvement on a five point Likert scale (“marked improvement” to “marked worsening”) and visual analogue scale (−100 mm=much worse, 0=same, 100=completely better). We reduced categorical data to success equating marked or moderate improvement.^19^

### Sample size

We based the sample size calculations on a clinically meaningful improvement of 15 mm on a 100 mm pain visual analogue scale for usual pain.^26^ ^29^ Assuming a standard deviation of 20 mm,^19^ a power of 0.80, and an α level of 0.01, we required 40 participants in each group. We increased the sample size by 10%, to 176 (44 in each group), to allow for loss to follow-up.

### Statistical analysis

Statistical analysis was done on a blinded, intention to treat basis using SPSS software (version 15.0). We chose the primary end points of 6, 12, and 52 weeks, as six weeks (immediately after the treatment period) could be considered to be the time of greatest effect, 12 weeks is a standard follow-up time in studies of patellofemoral pain syndrome,^30^ ^31^ ^32^ ^33^ and the long term (52 weeks) efficacy of foot orthoses or this specific physiotherapy programme has not been investigated. The dichotomous measure of success was expressed as relative risk reduction and numbers needed to treat. We analysed continuous outcome measures using univariate analysis of covariance, with baseline as a covariate and group allocation as a fixed factor. We included the characteristics of the participants and other baseline outcome measures as covariates in statistical models to determine their impact on outcome. Significance was set at 0.01 to accommodate the possibility of an inflated type I error rate resulting from multiple comparisons.

## Results

From May 2004 to May 2006, 1530 volunteers were screened and 179 enrolled in the study (fig 1). The trial was completed in June 2007, with 164 participants (92%) followed up at six weeks, 161 (90%) at 12 weeks, and 171 (96%) at 52 weeks. With the exception of duration, all groups were well matched at baseline (table 1). Including baseline data as covariates did not significantly influence outcomes.

**Figure fig1:**
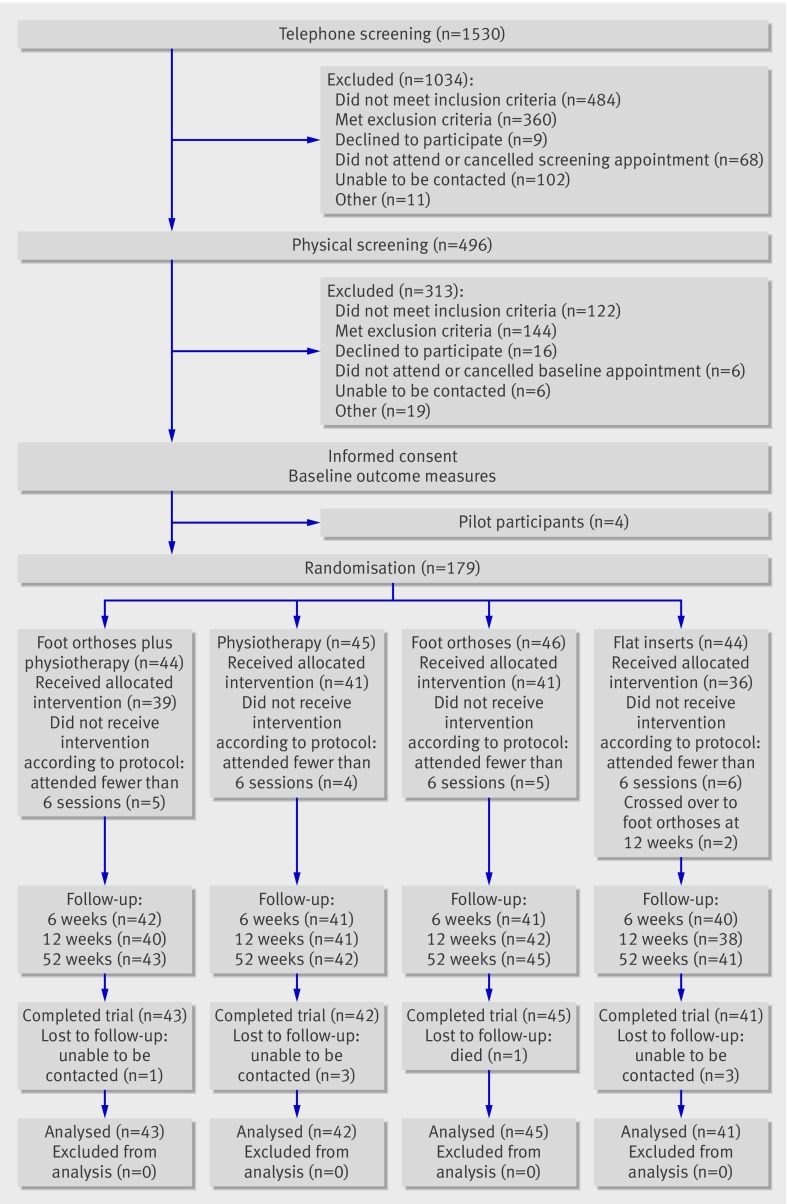
**Fig 1** Flow of participants through study

**Table 1 tbl1:** Baseline characteristics of participants for intervention groups and study cohort. Values are mean (SD) unless stated otherwise

Characteristics	Foot orthoses (n=46)	Flat inserts (n=44)	Physiotherapy (n=45)	Foot orthoses plus physiotherapy (n=44)	Total (n=179)
Age (years)	27.9 (5.3)	29 (6.0)	30.9 (5.8)	29.6 (5.6)	29.3 (5.8)
No (%) of women	25 (54.3)	20 (45.5)	29 (64.4)	26 (59.1)	100 (55.9)
Height (cm)	172.8 (9.1)	174.9 (10.5)	170.9 (8.4)	173.3 (9.6)	173 (9.5)
Weight (kg)	78.5 (20.4)	73.8 (15.9)	70.9 (14.6)	75.2 (22.3)	74.7 (18.6)
Body mass index	26.1 (5.6)	23.9 (3.5)	24.2 (4.7)	24.8 (6.2)	24.8 (5.1)
Physical activity* (kcal/kg)	40.6 (7.1)	41.3 (7)	41.2 (7.3)	40.8 (8.1)	41 (7.3)
No (%) with bilateral knee pain	26 (56.5)	25 (56.8)	26 (57.8)	25 (56.8)	102 (57)
Median (interquartile range) duration of knee pain (months)	42 (12.3-96)	24 (12-71)	37 (12.3-84.8)	24 (9-60)	28 (12-84)
Usual pain†	38.6 (16)	32.8 (15.1)	34.1 (17.0)	39.8 (17.6)	36.3 (16.6)
Worst pain†	59.4 (15.3)	56.6 (14.9)	61.4 (15.6)	64.8 (17.0)	60.5 (15.9)
Anterior knee pain scale‡	70.8 (9.0)	72.1 (9.3)	71.7 (11.3)	71.5 (9.8)	71.5 (9.8)
Functional index questionnaire§	10 (1.9)	10 (1.9)	10 (2.6)	9.3 (2.0)	9.8 (2.1)

*Physical activity over previous week questionnaire, total energy expended per day.

†Pain measured on 100 mm visual analogue scale; 0 mm=no pain, 100 mm=worst pain imaginable.

‡0-100 points; 100=no disability.

§0-16 points; 16=no disability.

Significant effects favoured foot orthoses over flat inserts at six weeks, with differences of 19.8 mm (99% confidence interval 4.0 to 35.6) on the continuous scale of global improvement, a number needed to treat of 4 (2 to 51) on the categorical scale (success equating marked and moderate improvement), and success rates of 85% (35/41) for foot orthoses and 58% (23/40) for flat inserts (fig 2, tables 2 and 3). These trends were mirrored when the categorical data on global improvement were collapsed to success equating marked improvement at six and 12 weeks. At six and 12 weeks no significant differences were found in global improvement between physiotherapy and foot orthoses, or between physiotherapy and combined physiotherapy and orthoses (tables 2 and 3). For each of the three a priori pairwise comparisons no significant differences were found between the groups on other outcome measures (table 3).

**Figure fig2:**
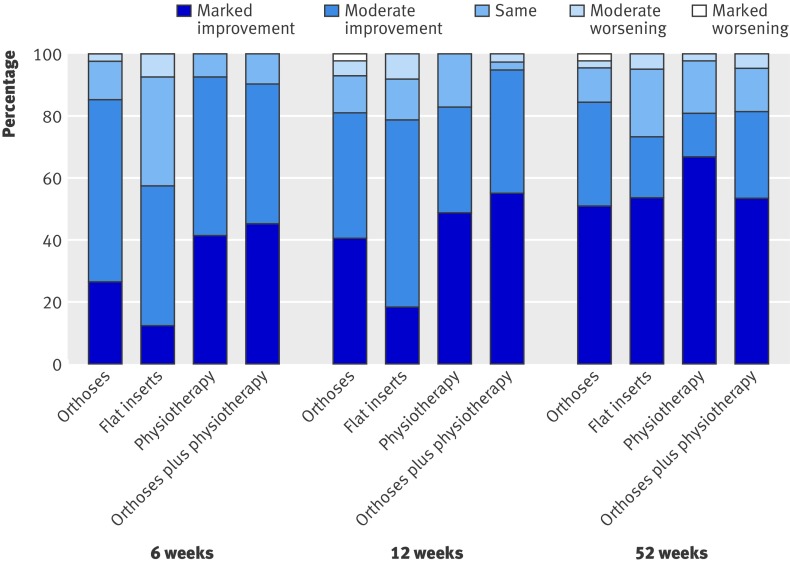
**Fig 2** Percentage of participants rating perceived improvement across categories from marked improvement to marked worsening

**Table 2 tbl2:** Absolute event rates of success of global effect and comparisons between groups for dichotomous measure of success expressed as relative risk reductions and numbers needed to treat (NNT)

Follow-up	No (%) moderately or markedly improved*				Between group differences (99% CI)†		
	Foot orthoses	Flat inserts	Physiotherapy	Foot orthoses plus physiotherapy	Foot orthoses *v* flat inserts	Physiotherapy *v* foot orthoses	Foot orthoses plus physiotherapy *v* physiotherapy
6 weeks	35/41 (85)	23/40 (58)	38/41 (93)	38/42 (90)	0.66 (0.05 to 1.17)‡; NNT 4 (2 to 51)‡†	0.5 (−0.84 to 1.85); NNT 14 (−8 to 4)	−0.3 (−2.83 to 2.24); NNT −45 (−5 to 6)
12 weeks	34/42 (81)	30/38 (79)	34/41 (83)	38/40 (95)	0.1 (−0.99 to 1.2); NNT 50 (−5 to 4)	0.1 (−1.06 to 1.25); NNT 51 (−5 to 4)	0.71 (−0.44 to 1.87); NNT 8 (−13 to 3)
52 weeks	38/45 (84)	30/41 (73)	34/42 (81)	35/43 (81)	0.42 (−0.43 to 1.24); NNT 9 (−9 to 3)	−0.22 (−1.6 to 1.14); NNT −29 (−4 to 6)	0.02 (−1.13 to 1.18); NNT 226 (−5 to 4)

*Participants rated improvement on five point Likert scale of global effect.

†Positive point estimate favours first listed condition.

‡Significant at P=0.01.

**Table 3 tbl3:** Mean (SD) scores and mean difference (99% confidence intervals) between groups for continuous primary outcome measures at 6, 12, and 52 weeks (adjusted for baseline), according to intervention for patellofemoral pain syndrome

Variables	Mean (SD) for each group					Mean (99% CI) differences between groups		
	Foot orthoses	Flat inserts	Physiotherapy	Foot orthoses plus physiotherapy		Foot orthoses *v* flat inserts	Physiotherapy *v* foot orthoses	Foot orthoses plus physiotherapy *v* physiotherapy
Global improvement (−100-100)*:								
6 weeks	37.6 (27.2)	17.8 (27.2)	45.4 (27.2)	48.7 (27.2)		19.8 (4.0 to 35.6)†	7.8 (−7.8 to 23.5)	3.2 (−12.3 to 18.8)
12 weeks	46.7 (32.8)	30.6 (32.8)	53.4 (32.8)	61.8 (32.8)		16.1 (−3.0 to 35.3)	6.7 (−12.1 to 25.5)	8.4 (−10.7 to 27.4)
52 weeks	52.3 (39.8)	49.9 (39.8)	54.7 (39.8)	55.2 (39.8)		2.4 (−20.0 to 24.8)	2.4 (−19.9 to 24.7)	0.5 (−22.0 to 23.0)
Usual pain (0-100 mm)‡:								
6 weeks	25.4 (17.4)	33.4 (17.5)	21.2 (17.3)	19.4 (17.4)		−8 (−18.1 to 2.1)	−4.2 (−14.2 to 5.8)	−1.8 (−11.8 to 8.2)
12 weeks	22.1 (17.8)	24.5 (18)	20.1 (17.8)	16.4 (17.9)		−2.4 (−12.9 to 8.1)	−2 (−12.2 to 8.2)	−3.7 (−14.1 to 6.6)
52 weeks	16.2 (18.5)	17.9 (18.6)	13.9 (18.5)	14.4 (18.6)		−1.7 (−12.2 to 8.8)	−2.2 (−12.6 to 8.1)	0.4 (−10.1 to 10.9)
Worst pain (0-100 mm)‡:								
6 weeks	39.8 (21.7)	48 (21.8)	32.2 (21.6)	28.5 (21.9)		−8.1 (−20.7 to 4.4)	−7.7 (−20.2 to 4.8)	−3.6 (−16.0 to 8.8)
12 weeks	33.3 (22.2)	35 (22.4)	26.8 (22.2)	26.5 (22.3)		−1.7 (−14.7 to 11.3)	−6.5 (−19.2 to 6.2)	−0.2 (−13.1 to 12.7)
52 weeks	27.6 (23.7)	26.1 (23.9)	22.2 (23.7)	18.8 (23.9)		1.5 (−11.9 to 15.0)	−5.5 (−18.8 to 7.9)	−3.3 (−16.8 to 10.1)
Anterior knee pain scale (0-100)*:								
6 weeks	79.7 (9.1)	74.8 (9.1)	83.4 (9.1)	83.6 (9.1)		4.9 (−0.4 to 10.2)	3.7 (−1.6 to 9.0)	0.2 (−5.0 to 5.5)
12 weeks	81.8 (9.9)	80.9 (9.9)	84.9 (9.9)	86.7 (9.9)		0.9 (−4.9 to 6.6)	3.1 (−2.5 to 8.8)	1.8 (−4.0 to 7.5)
52 weeks	85.5 (9.7)	86.9 (9.7)	87.9 (9.7)	91.5 (9.7)		−1.5 (−7.3 to 4.4)	2.5 (−3.3 to 8.2)	3.6 (−2.5 to 9.7)
Functional index questionnaire (0-16)*:								
6 weeks	11.8 (2.3)	11.1 (2.3)	12.9 (2.3)	13.3 (2.3)		0.7 (−0.6 to 2.0)	1.0 (−0.3 to 2.3)	0.5 (−0.8 to 1.8)
12 weeks	12.3 (2.3)	12.0 (2.3)	13.3 (2.3)	13.9 (2.3)		0.2 (−1.1 to 1.6)	1.0 (−0.3 to 2.4)	0.6 (−0.8 to 1.9)
52 weeks	13.0 (2.6)	13.4 (2.6)	14.2 (2.6)	13.8 (2.6)		−0.5 (−1.9 to 1.0)	1.3 (−0.2 to 2.7)	−0.5 (−1.9 to 1.0)

*Positive score favours reference group (first group listed in comparison).

†Significant at P=0.01.

‡Negative score favours reference group.

Over 52 weeks all groups had clinically meaningful improvements in worst pain severity (>20 mm on pain visual analogue scale), anterior knee pain scale (>10 points), and functional index questionnaire (>2 points; tables 1 and 3).^26^ Three of the four groups (foot orthoses, physiotherapy, foot orthoses plus physiotherapy) also had clinically meaningful improvements in usual pain severity, whereas the improvement in usual pain for the group receiving flat inserts was slightly less than 20 mm. No significant differences were found between groups on any primary measure at 52 weeks.

### Cointerventions

Table 4 outlines the participants’ use of cointerventions. No significant differences were found in reported rates of use between foot orthoses and flat inserts (14/40, 35% *v *15/39, 38%; relative risk reduction 0.09, 99% confidence interval −0.6 to 0.76), physiotherapy and foot orthoses (16/43, 37% *v* 14/40, 35%; −0.06, −0.78 to 0.68), or foot orthoses plus physiotherapy and physiotherapy alone (9/40, 23% *v* 16/43, 37%; 0.4, −0.3 to 1.01). Two participants assigned to flat inserts crossed over to foot orthoses after 12 weeks.

**Table 4 tbl4:** Participant reported use of cointerventions for patellofemoral pain during trial*

Additional intervention	Foot orthoses (n=40)	Flat inserts (n=39)	Physiotherapy (n=43)	Foot orthoses plus physiotherapy (n=40)	Total (n=162)
None	26	24	27	31	108
General practitioner or specialist	3	0	0	1	4
Physiotherapy	3	1	1	2	7
Foot orthoses	0	3†	1	0	4
Massage	1	0	1	0	2
Over counter drugs‡	11	12	13	7	43
Prescription drugs‡	0	1	0	0	1
Acupuncture	1	0	0	1	2
Complementary medicine	1	2	3	2	8
Heat rub	1	0	1	1	3
Ice or heat	4	0	1	1	6
Knee brace	1	0	0	1	2

*Some participants used more than one additional intervention.

†Includes two participants who crossed over to receive foot orthoses after 12 weeks as recommended by trial physiotherapists.

‡Analgesics and non-steroidal anti-inflammatory drugs.

### Side effects

A greater proportion of participants reported mild side effects with the foot orthoses (foot orthoses 31/43, 72%; foot orthoses plus physiotherapy 20/41, 49%) than with the flat inserts (15/39, 38%; relative risk reduction −0.58, 99% confidence interval −1.01 to −0.09). These consisted of rubbing and blistering, discomfort, and pain in the toes, feet, and ankles, which on the whole responded to increasing wear and minor adjustments to the orthoses (for example, heat moulding and additions) and did not prevent wearing of the orthoses or inserts. Thirty four participants (physiotherapy 18/44, 41%; foot orthoses plus physiotherapy 16/41, 39%; relative risk reduction 0.05, −0.59 to 0.67) reported a reaction to daily patellar taping (for example, skin irritation, blistering). Two participants (physiotherapy group and foot orthoses group) experienced low back pain that required additional physiotherapy.

## Discussion

Foot orthoses produced short term improvements beyond that of flat inserts, with the number needed to treat indicating that four patients would need to be treated with orthoses to have one additional patient experience improvement in patellofemoral pain. Foot orthoses were similar in effect to physiotherapy, and combining foot orthoses with physiotherapy did not provide additional improvement beyond physiotherapy alone. In the long term, clinically meaningful improvements occurred in pain and function for all interventions but no differences were found between interventions. The overall pattern of effect implies that foot orthoses and physiotherapy each hasten resolution of the condition, which is an important benefit for a common, chronic condition.

Treatment costs are a further consideration for practitioners and their patients. Assuming recommended retail pricing, in addition to consultation fees (usually between two to four consultations), the orthoses (three pairs, including additions) would cost $A174 (£79; €100) compared with $A45 for three flat inserts. This would seem to be a reasonable alternative to physiotherapy (six sessions at around $A495, including tape). A cost benefit analysis is required to investigate this further.

The interventions used in this trial, including the flat inserts, produced only mild side effects in the early phase of treatment. Despite the orthoses having relatively more minor side effects than the flat inserts, they showed a greater improvement in the first six weeks, suggesting that these side effects did not adversely influence treatment outcomes. About 40% of participants who received the physiotherapy intervention (with or without foot orthoses) experienced skin reactions with daily taping of the patella, despite exclusion of participants with known allergies to tape. It is difficult to know whether this is abnormally high due to inadequate reporting in previous trials, but should be considered in clinical application.

The prescription of foot orthoses for musculoskeletal pain is characterised by a lack of evidence from high quality clinical trials.^15^ ^20^ Our study provided level II evidence for the use of foot orthoses in patellofemoral pain syndrome. Our data corroborate findings from a smaller study of 20 adolescent females aged 13 to 17, which found statistically significant improvements in pain during gait, sitting, and squatting after eight weeks of treatment.^21^ The authors did not supply point estimates of effect. Furthermore, the magnitude of the effect of physiotherapy that we observed on primary outcome measures at six weeks was comparable with that of another study.^19^

Some authors contend that the contoured form of foot orthoses is critical for controlling foot motion, usually excessive pronation.^34^ ^35^ ^36^ This exists despite three key issues, all of which impinge on the conduct of a randomised controlled trial. Firstly, research shows generally equivocal and non-systematic effects of the ability of foot orthoses to control motion.^37^ ^38^ ^39^ Secondly, alternative means may be available by which foot orthoses exert clinical effects, such as by serving as space fillers to facilitate full plantar contact,^40^ which some regard to be clinically beneficial.^41^ ^42^ Thirdly, previous research has failed to show that people with patellofemoral pain syndrome have excessive foot pronation compared with controls.^10^ ^43^ On the basis of these issues, we included in our randomised controlled trial a flat shoe insert to evaluate the clinical efficacy of the contoured form of foot orthoses.^25^ Our findings of a clinically beneficial effect in favour of the contoured orthoses provides a solid foundation on which to consider the mechanisms of action of foot orthoses and plan future research.

Point estimates of effect between foot orthoses and flat inserts were detected by using measures of global improvement, but not by using measures of pain or physical function, even though these measures were sensitive to change over time within each group. This reflects the moderate correlations between global improvement rating scales and these measures of pain and function reported by researchers^26^ in their evaluation of outcome measures used in their randomised controlled trial.^19^ They recommended that clinical trials of patellofemoral pain syndrome incorporate a measure of perceived global response to treatment, largely on the basis that this scale feasibly encompasses many dimensions of patellofemoral pain syndrome that are meaningful to the patient (for example, pain, function, disability, participation, psychosocial factors). It is likely that a rating of global improvement captures more comprehensively the patient experience, a notion that requires further exploration to understand better the clinical relevance.

### Strengths and limitations

The prescription of foot orthoses for musculoskeletal pain is characterised by a lack of solid evidence from quality clinical trials.^20^ We studied the long term efficacy of foot orthoses in the management of patellofemoral pain syndrome. This is a clinically important issue as the condition is highly prevalent and foot orthoses are prescribed worldwide. We incorporated the recommendations from the consolidated standards of reporting trials into the methodological design, which further strengthens the validity of findings.^44^ Importantly, the attrition rate was low, with 8% of primary outcome data missing at six weeks, 10% at 12 weeks, and 4% at 52 weeks.

Unlike other clinical trials, we did not select those treated with foot orthoses on the basis of foot posture (for example, excessive pronation^22^), largely because no valid method currently exists to identify a priori those who may benefit from foot orthoses. It is possible that participants fitted with orthoses in our trial were (randomly) heterogeneous for foot posture, yet we still found small but beneficial effects of prescribing foot orthoses compared with flat inserts. Conceivably, if the classification of patients becomes possible,^45^ then the point estimates of effect we report are likely to be an underestimate.

The characteristics of the participants in our trial were similar to those reported by others for age, height, sex, proportion with bilateral patellofemoral pain syndrome, duration of condition, severity of pain, anterior knee pain scale, and functional index questionnaire.^10^ ^19^ ^30^ ^33^ Feasibly this represents the broader population of patients with patellofemoral pain syndrome who visit general medical practices and strengthens the external validity of the findings of our study. Further reinforcing the external validity of our findings we used physiotherapists from primary care practices in the community, and with only a short duration of training in the protocol (about 1.5 days) they were able to successfully implement an effective foot orthosis intervention, which had a similar effect to the multimodal physiotherapy programme.

A limitation of this study is the number of comparisons between groups. Although we used 99% confidence limit to assist in control of type I errors, it is possible that the significant finding between foot orthoses and flat inserts was due to chance. Notwithstanding this, a number needed to treat of 4 could be regarded as a clinically meaningful effect and in part counters the possibility of a type I error in the comparison of orthoses with flat inserts at six weeks.

A further limitation was that we did not include a control group for clinical course so we cannot decisively conclude that foot orthoses or physiotherapy were better than no treatment over 52 weeks. Nevertheless, a case may be made for intervening with foot orthoses or physiotherapy as over 80% of participants in our study were improved at 52 weeks, compared with 50% of participants followed up at four years in a prospective long term study of the clinical course of patellofemoral pain syndrome.^11^

### Conclusions

Prefabricated foot orthoses are superior to flat inserts in the short term management of patellofemoral pain syndrome, implying that their contoured shape is therapeutic. We found no differences between the effects of foot orthoses and physiotherapy, nor was there any benefit of adding foot orthoses to physiotherapy. Considering that all treatment groups showed clinically meaningful long term improvements, general practitioners may seek to hasten recovery by prescribing foot orthoses.

**What is already known on this topic**

Patellofemoral pain syndrome is highly prevalent in sports medicine and presents often to general practices
Foot orthoses are often prescribed despite a lack of evidence highlighted by systematic reviews

**What this study adds**

Foot orthoses produce earlier and larger improvements in patellofemoral pain syndrome than flat inserts
Adding foot orthoses to physiotherapy does not improve physiotherapy outcomes

## Notes

We thank Vasyli International for providing the foot orthoses and flat inserts; Ausmedic and Access Health for supplying concessions on the purchase of equipment used in the physiotherapy interventions; the project physiotherapists and their practice staff; Jenny McConnell for her assistance in running the protocol workshops; and research assistants Jane Buckley, Bula Elwell, and Erica Williams.

Contributors: NC recruited and screened the participants, carried out the baseline and follow-up outcome measures and data entry and analysis, and prepared the manuscript. KC was involved in the methodological design and preparation of the manuscript. EB assisted in the trial design and carried out the randomisation procedures. RD advised on the statistical design of the trial and data analysis and interpretation. TM was involved in the National Health and Medical Research Council grant application and trial design and reviewed the manuscript. BV, in his capacity as sole chief investigator on the National Health and Medical Research Council grant, supervised the conduct of the trial, the suitability of included participants, data analysis, and preparation of the manuscript, and is guarantor.

Ethical approval: University of Queensland’s medical research ethics committee.

Provenance and peer review: Not commissioned; externally peer reviewed.

## Notes

**Cite this as: ***BMJ* 2008;337:a1735
